# Impact of matrix metalloproteinase-8 gene variations on the risk of thoracic aortic dissection in a Chinese Han population

**DOI:** 10.1007/s11033-013-2704-2

**Published:** 2013-09-25

**Authors:** Xiao-Zeng Wang, Xiao-Mo Du, Quan-Min Jing, Xing-Xing Li, Ruo-Xi Gu, Jiao Wang, Ya-Ling Han

**Affiliations:** Department of Cardiology, Cardiovascular Research Institute, Northern Hospital, 83 Wenhua Road, Shenyang, 110840 Liaoning China

**Keywords:** Thoracic aortic dissection, MMP8, Polymorphism

## Abstract

The importance of matrix metalloproteinase 8 (MMP8) expression during the progression of thoracic aortic dissection (TAD) has been recently emphasized. Genetic variations that affect proteinase expression or activity might contribute to the pathogenesis of TAD. In this study, we investigated whether the *MMP8* C-799T genotype is associated with TAD. The frequency distributions of the *MMP8* C-799T polymorphism were determined by direct sequencing. Associations between the polymorphism and disease progression in TAD were investigated. The level of plasma and tissue MMP8 was measured by enzyme-linked immunosorbent assay and western blotting. The *MMP8* C-799T polymorphism was significantly associated with susceptibility to disease progression in TAD patients (*n* = 152) than in controls (*n* = 147) (*P* = 0.004, OR = 0.62, 95 % CI 0.45–0.86). The TT homozygotes had a significantly higher risk of TAD compared to C allele carriers in a logistic regression model, after adjustment for the conventional risk factors for TAD. The plasma MMP8 concentration was significantly higher in TAD patients compared to control patients (*P* < 0.05). TT genotypes had increased MMP8 levels compared to CC and CT genotype carriers in both TAD and control subjects (*P* < 0.05). The C-799T polymorphism in the *MMP8* promoter is part of the genetic variation underlying the susceptibility of individuals to the progression of TAD.

## Introduction

Thoracic aortic dissection (TAD) is a life-threatening emergency associated with a high risk of mortality and morbidity [1, 2]. Chronic inflammation, increased neoangiogenesis, enhanced oxidative stress, and extracellular matrix (ECM) degradation involved in the pathological process of TAD. Damage in the lesion structure of the aortic vascular wall could be observed in TAD patients, it was also can be observed infiltration of inflammatory cells surrounding the location of the intimal entry tear.

Vascular smooth muscle cells (VSMCs) and ECM proteins play an important role in the pathological process of TAD, and provide the bulk of the medial layer of the aorta [3–5]. Maintaining a balanced composition of VSMCs and ECM proteins appears to be critical for preserving the important functional role of the thoracic aorta [6, 7]. It is well known that pathological remodeling of the aorta and excessive ECM degradation may lead to progressive aortic wall rupture [8].

Matrix metalloproteinases (MMPs) are a family of more than 20 zinc-dependent proteolytic enzymes, which play vital roles in TAD related to ECM degradation [9, 10]. MMP8 is synthesized predominantly by cells of the neutrophil lineage, and has been found to be released upon stimulation of other cell types [11]. Recent studies have shown that excessive activation of MMP8 might contribute to rapid aortic expansion and rupture [12]. MMP8 may contribute to TAD by causing the degradation of collagens within the aortic wall, leading to expansion and rupture of the aortic wall. *MMP8* is located on chromosome 11p22.3 and consists of 10 primary exons. Data from leukocyte cells suggests that *MMP8* promoter C-799T variant has increased activity compared to a promoter containing wild-type alleles [13, 14]. The present study was designed to analyze genetic variation in *MMP8* and assess its genetic association with TAD in Han Chinese, and to add knowledge to the prevention and treatment of TAD.

## Materials and methods

### Study subjects

A total of 152 patients with TAD and 147 controls were recruited from the Shenyang Northern Hospital during the period March 2002 to August 2011. The TAD diagnosis was confirmed by noninvasive imaging such as transesophageal echocardiography, helical computed tomography (CT), or magnetic resonance imaging (MRI) 0.147 age- and gender-matched healthy subjects were used as controls. A complete clinical history was obtained from all subjects. Patients with a history of hematologic, neoplastic, renal, liver, or thyroid disease were excluded. Additionally, patients who had inflammatory, infectious or autoimmune diseases, type-1 diabetes mellitus, or familial hyperlipidemia were also excluded. The study was performed with the approval of the ethics committee in Shenyang Northern Hospital.

To further investigate the impact of gene polymorphisms on pathological phenotypes, the levels of plasma MMP8 were determined in a subset population selected from our original cohort between January 2008 and May 2011. Control blood samples were collected from 60 healthy subjects.

### Tissue collection

Aortic tissue was obtained intraoperatively, from consenting patients undergoing open repair of their aneurysm or bypass of their occluded aorta. Autopsy specimens from patients who died of unrelated causes were used as the control tissue. The aortic specimen was immediately cut into 3 mm cubes, snap-frozen in liquid nitrogen and stored at −80 °C for protein extraction. All protocols using human samples were approved by the Shenyang Northern Hospital, and all samples were obtained with written informed consent. For some TAD patients who died suddenly soon after admission, or who were diagnosed at autopsy, consent was obtained from family members.

### Genotyping

Genomic DNA was extracted from the white-cell pellet obtained from blood, using a modified salt-extraction methods (Tiangen Biotech Co. Ltd., Beijing, China) following the manufacturer’s instruction [15]. The *MMP8* C-799T polymorphism was analyzed in our case–control population by direct PCR sequencing after extraction of genomic DNA from blood leukocytes. The primers used were forward 5′TCCCTAGTCTATAAGTTAGA3′ and reverse 3′CCTGTGAATATAAGCCAAAG5′. PCR cycling conditions were as follows: an initial denaturation at 94 °C for 4 min, followed by 35 cycles at 94 °C for 30 s, 56 °C for 30 s, and 72 °C for 30 s. PCR reactions were performed in a Bio-Rad thermal cycler and in a final volume of 50 μL. DNA sequencing was performed using the ABI Prism 3730 genetic analyzer with the ABI dye terminator cycle sequencing kit. Genotypes were determined by independent investigators who were blinded to patients’ identities and phenotypes.

### Determination of plasma MMP8 levels

Blood samples for the determination of MMP8 plasma levels were collected on ice in tubes containing EDTA and aprotinin, and were centrifuged at 3,000×*g* for 5 min at 4 °C to isolate plasma. Plasma MMP8 levels were measured using the human MMP8 Quantikine ELISA Kit (R&D Systems, Minneapolis, MN, USA) according to the manufacturer’s instructions. All samples were analyzed twice, and mean values were used for further statistical analysis.

### Western blotting

MMP8 was extracted from pulverized tissue using a protein extraction buffer containing 0.1 % Triton X-100 and 0.2 ml protease inhibitor cocktail (Set III, Sigma) in PBS pH 7.4. After centrifugation at 15,000×*g* for 10 min, the supernatant was used for Western blotting. Total cell protein concentrations were determined using the DC Protein Assay Kit (Bio-Rad). Proteins were resolved by sodium dodecyl sulfate–polyacrylamide gel electrophoresis, and transferred to polyvinylidene fluoride membranes. The membranes were incubated with appropriate primary antibodies. Specific binding was detected using HRP conjugated secondary antibodies and an ECL western blotting Kit. The blots were quantified using a Bio-Rad gel documentation system.

### Statistical analysis

Hardy–Weinberg equilibrium was assessed in the controls using the Chi square test. Statistical analysis was performed using SPSS 15.0 (SPSS Inc., Chicago, IL). Allele frequencies were determined by gene counting. Tests were used to examine the differences in allele frequencies and genotype distributions between the groups. Multivariable logistic regression analysis was performed to adjust for the risk factors age, sex, and hypertension. The association between genotyped polymorphisms and risk of disease was estimated by *P* values, odds ratios (ORs), and 95 % confidence intervals (95 % CIs). One-way analysis of variance (ANOVA) was used to analyze plasma levels of MMP8 between the TAD patient group and the control group. A *P* value of <0.05 was considered statistically significant.

## Results

### Characteristics of the study subjects

The main baseline characteristics of patients with TAD and controls are shown in Table 1. Although an effort was made to obtain a good match in age and sex between controls and TAD patients, there were more male patients in the TAD group (2:1) than in the control group in our population. The significant difference in smoking consumption status (*P* < 0.01) between the TAD and control groups may be owing to the difference in sex ratio, because fewer women smoke in China.

**Table 1:** Demographic and clinical characteristics of the study participant

	TAD (*n* = 152)	Control subjects (*n* = 147)
Age (mean ± SD)	51.4 ± 8.9	50.2 ± 9.4
Gender distribution (M/F)	112 (40)	106 (41)
Type A	43	
Type B	109	
Smoker, *n* (%)	75 (49.3)	21 (14.2)
Hypertension, *n* (%)	106 (69.7)	15 (10.2)
Diabetes mellitus, *n* (%)	19 (12.5)	13 (8.8)
Acute renal failure, *n* (%)	12 (7.8)	–
Limb ishemia, *n* (%)	–	–
Serum measurement		
TG (mmol/L)	2.12 ± 1.43	1.82 ± 1.34
TC (mmol/L)	4.83 ± 1.34	4.46 ± 0.98
LDL-C (mmol/L)	2.85 ± 0.89	2.34 ± 0.75
HDL-C (mmol/L)	1.52 ± 0.36	1.43 ± 0.28
Stain, *n* (%)	46 (30.3)	14 (9.5)
β-blocker, *n* (%)	148 (97.4)	13 (8.8)
ACE-inhibitor, *n* (%)	132 (86.8)	18 (12.3)

Values for continuous variables are expressed as mean ± S.D. Figures in parentheses are percentages

*TAD* thoracic aortic dissection, *HDL-C* high density lipoprotein cholesterol, *LDL-C* low density lipoprotein cholesterol, *TC* total cholesterol; *TG* triglyceride

### Association of genotype with phenotype

Genotype and allele frequencies of the *MMP8* gene polymorphisms are shown in Table 2. The minor allele frequencies of the polymorphisms were similar to the reported frequencies in the Han Chinese population from the International HapMap Project. There was a significant difference between patients with TAD and controls for the *MMP8* C-799T polymorphism (*P* = 0.008, Table 2). The T allele frequency was significantly higher in TAD patients than in controls (*P* = 0.004, OR = 0.62, 95 % CI: 0.45–0.86).On the basis of multivariable logistic regression analysis with adjustment for cardiovascular risk factors; subjects bearing the TT homozygotes had significantly increased susceptibility to TAD compared to CT and CC allele carriers (*P* < 0.05).

**Table 2:** Genotypic and allelic frequencies of *MMP*-*8* -C799T polymorphism in TAD patients and control subjects

Tag SNP	Genotype	Control subjects	TAD subjects	*P* value	OR	95 % CI
rs11225395	CC	57 (38.8)	45 (29.6)	0.008		
	CT	71 (48.3)	66 (43.4)			
	TT	19 (12.9)	41 (27.0)			
	C	185 (62.9)	156 (51.3)	0.004	0.62	(0.45–0.86)
	T	113 (37.1)	148 (48.7)			

*P* values were obtained by *χ* ^2^-test. *P* < 0.05

*OR* odds ratio; 95 % *CI* 95 % confidence interval

### *MMP8* levels in TAD patients and controls

**Fig. 1:**
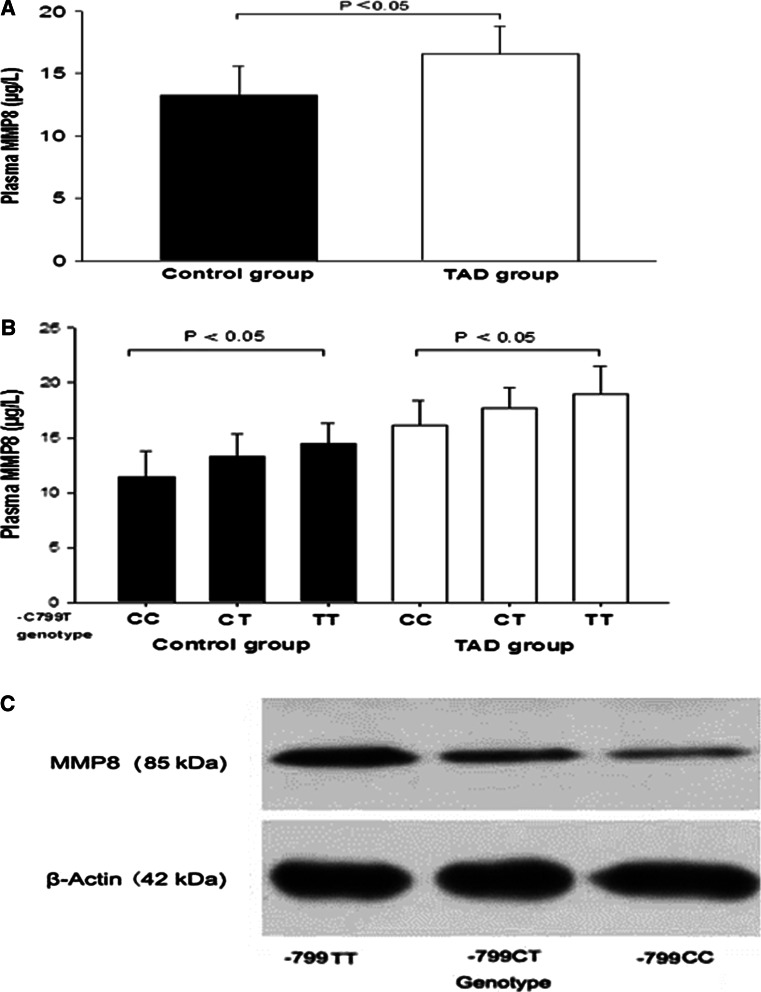
Western blot and ELISA Analyses for MMP8 in control and TAD subjects. Plasma MMP8 levels in TAD patients and control subjects by ELISA (**a**); Plasma MMP8 levels in subgroup subjects according to MMP8 -C799T genotype (**b**); MMP8 protein concentration in the TAD wall of patients having either the TT, CT or CC genotypes (**c**).The difference between groups was analyzed by one-way ANOVA tests

Figure 1a shows the effect of the *MMP8* C-799T polymorphism on MMP8 activity in controls and TAD patients. There was a significant difference in plasma MMP8 levels among 3 genotypes of C-799T in the control and TAD groups (*P* < 0.05). MMP8 activity in TAD patients ranged from 13.90 to 22.69 μg/L, and from 8.16 to 18.73 μg/L in controls. We found that the MMP8 level was significantly higher in subjects with the TT genotype (19.01 ± 2.50 μg/L) than those with CT (17.66 ± 1.92 μg/L) and CC (16.14 ± 2.26 μg/L) genotypes in the TAD group (*P* < 0.05) (Fig. 1b). Western blotting analysis showed that significantly greater amounts of MMP8 protein were present in the thoracic aorta wall of *MMP8* 799TT homozygotes, than in *MMP8* C-799T heterozygotes and *MMP8* 799CC homozygotes (Fig. 1c).

## Discussion

The idea of an intrinsic rupture of the aortic wall as the cause of TAD has been put forward in recent years. Decreased elasticity as a result of elastin degradation/rupture is an early event in the procession of TAD [1, 2, 16–18]. MMP8, with the ability to cleave the native interstitial collagen, plays vital roles in TAD related to ECM metabolism and aortic wall remodeling. In addition, TADs group contain significantly higher MMP8 level in plasma than control group [11, 12].

In the present study, we found that the *MMP8* C-799T polymorphism is associated with increased susceptibility to disease progression in TAD patients. Furthermore, enzyme-linked immunosorbent assay and western blotting analysis showed increased MMP8 production in TAD patients with TT carriers. To the best of the authors’ knowledge, this is the first report showing that naturally occurring genetic variants of the *MMP8* gene may indicate an individual’s susceptibility to TAD. Because the C-799T polymorphism is located in the *MMP8* promoter, functional analysis of the *MMP8* C-799T variation indicates that it has an effect on *MMP8* promoter activity, with the T allele having a greater effect on driving gene expression, and which affect MMP8 function indirectly.

Because the T allele is associated with higher promoter activity than the C allele, it is possible that the nuclear protein(s) selectively binding to the T allele is a transcriptional enhancer [19, 20]. The *MMP8* C-799T polymorphism may alter the binding affinity of the nuclear protein(s) and affect the regulation of MMP8 expression. Additional molecular functional analysis of the *MMP8* C-799T polymorphism is necessary to elucidate the underlying mechanism of increased MMP8 promoter activity.

Although all patients recruited in this study underwent noninvasive imaging, and were recruited on clinical criteria, we recognize there are a number of important caveats that must be kept in mind when interpreting the results of association studies. First, we analyzed the association between the *MMP8* C-799T polymorphism and TAD, while other single nucleotide polymorphisms (SNPs) linked with the *MMP8* C-799T polymorphism were not investigated. Therefore, we cannot rule out the presence of another SNP or mutation in linkage disequilibrium with the C-799T polymorphism. Second, the study population for this analysis consisted of northern Chinese Han people only, thus the results of this study may not be applicable to other racial or ethnic groups. Additional larger population-based case–control studies are necessary to understand the association between the genetic variation and TAD in the *MMP8* loci. Third, the male to female ratio in patients and control individuals differed, and this statistical difference may suggest a sex-specific effect.

In conclusion, our results provide further evidence that the *MMP8* C-799T polymorphism is associated with TAD in a Chinese Han population. The minor allele is associated with decreased MMP8 levels in both TAD patients and control subjects. These results suggest that C-799T polymorphism in the *MMP8* gene is a novel genetic risk marker for TAD in Han Chinese. Additional studies in other populations from various ethnic groups are needed to confirm the present findings, before the importance of the *MMP8* C-799T polymorphism in TAD risk can be fully ascertained.

## Abbreviations

MMP8: Matrix metalloproteinase 8
TAD: Thoracic aortic dissection
CI: Confidence interval
OR: Odds ratio
PCR: Polymerase chain reaction
SNP: Single nucleotide polymorphism
